# Prevalence and factors associated with adverse drug reactions among heart failure patients hospitalized at Mbarara Regional Referral Hospital, Uganda

**DOI:** 10.1186/s12872-022-02937-7

**Published:** 2022-11-11

**Authors:** Efrata Ashuro Shegena, Konjit Abebe Nigussie, Robert Tamukong, Boniface Amanee Elias Lumori, Tadele Mekuriya Yadesa

**Affiliations:** 1 Department of Pharmacy, Mbarara University of Science and Technology, Mbarara, Uganda; 2 Pharmacy Biotechnology and Traditional Medicine Center, Mbarara University of Science and Technology, Mbarara, Uganda; 3 Department of Internal Medicine, Mbarara University of Science and Technology, Mbarara, Uganda; 4 Department of Pharmacy, Ambo University, Ambo, Ethiopia

**Keywords:** Heart failure, Adverse drug reaction, Severity, Preventability

## Abstract

**Background:**

Adverse drug reaction (ADR) of medications remains an obstacle to achieving optimal disease outcomes. This study aimed to assess the prevalence and associated factors of ADR among Heart failure (HF) patients hospitalized at Mbarara Regional and Referral Hospital.

**Method:**

A prospective observational study was conducted among hospitalized HF patients from November 2021 to January 2022. Univariate and multivariate logistic regression was employed to determine factors associated with the ADR.

**Result:**

Overall, 118 HF patients were included in the study with a median age of 43 years. A total of 164 ADRs were identified during the follow-up period of 1011 days. The incidence of new ADRs was 106 ADRs/1000 person-days. The prevalence of ADR was 59.3%. Of the 164 ADRs, 118(71.9%) were probable. The gastrointestinal system was the most frequently (27.5%) affected system. Over half (86, 52.4%) of the ADRs were mild and 96(58.5%) were preventable. Age group 19–59(AOR 0.15[0.03–0.35] at 95%CI, *p* = 0.013), herbal use (AOR 3.07[1.01–9.32] at 95%CI, *p* = 0.048), poly-pharmacy (AOR 8.7[2.4–15.77] at 95%CI, *p* < 0.001) and drug-drug interaction (AOR 6.06[2.79–12.5] at 95%CI, *p* = 0.004) were significantly associated with ADRs among HF patients.

**Conclusion:**

More than half of the hospitalized HF patients experienced at least one ADR during their hospital stay. The use of herbal medicines, poly-pharmacy, and drug-drug interaction were associated with a high risk of ARDs whereas the age group 19–59 years was less likely to experience ADRs.

## Introduction

### Background

Non-communicable diseases (NCDs) are the leading cause of death globally contributing to 73.4% of all deaths. Cardiovascular diseases (CVDs) are the leading of all NCDs being responsible for approximately 31% of all deaths [1, 2]. Cardiovascular diseases (CVDs), contribute to 17.3 million deaths per year worldwide and are expected to increase to 23.6 million approximately by 2030 [3].

Forty-four percent of patients with newly diagnosed CVD have heart failure (HF) [4]. This rate seems to be declining in developed nations, but in Sub–Saharan Africa (SSA), it is rather progressively taking over infectious diseases as the leading cause of hospitalization, morbidity, and premature mortality [5]. A recent study on the epidemiology of HF estimates that 64.3 million people have HF worldwide [6]. The burden of HF is high in Low and Middle-income countries (LMIC) due to its high impact on the young working class, and the associated high mortality rate [7, 8].

The management of HF mainly relies on lifelong therapy with multiple medications [9, 10]. According to current updated guidelines, Angiotensin converting enzyme inhibitor (ACEI)/ Angiotensin receptor blocker (ARB)/ Angiotensin receptor-neprilysin inhibitor (ARNI), Mineralocorticoid antagonists (MRA), β-Blockers, Sodium-glucose co-transporter 2 inhibitors (SGLT2 inhibitor) and loop diuretics are the initial medications recommended for the treatment of HF [11, 12, 13].

The benefits of these medications in slowing HF progression, reducing morbidity and mortality, and/or improving symptoms are clearly established [14]. However, Adverse drug reactions to the medications remain an obstacle to achieving optimal disease outcomes in the management of HF across the world [9, 10].

Adverse drug reaction (ADR) is defined as “an appreciably harmful or unpleasant reaction, resulting from an intervention related to the use of a medicinal product, which predicts hazard from future administration and warrants prevention or specific treatment, or alteration of the dosage regimen, or withdrawal of the product” [15].

The common ADRs in HF patients have been linked with the medications used for treating the underlying cause and comorbid diseases. Factors contributing to the development of ADR could be drug-related, disease-related, or patient-related. Age, gender and disease states are patient-related contributing factors in the development of ADR among HF patients [16, 17]. Polypharmacy is the most important drug-related predisposing factor for ADRs; HF patients being the most vulnerable [16, 17, 18]. Inappropriate prescriptions or drug-drug interactions from poly-pharmacy were significant risks for ADR [19, 20]. Not only medications but the comorbid condition of a patient has an impact on HF patients [18, 21].

Adverse drug reactions entail a significant direct impact on a patient’s health status and burden on healthcare facilities [22]. ADR has an economic impact in that it extends hospital stay, adds clinical investigation in serious cases, and imposes costly management of ADRs in hospitalized patients [9, 23, 24]. Retrospective studies done in China [25], Italy [26], and Germany [27] showed cost associated with the management of ADR was €40.8, €585, and €970, per patient respectively.

In Uganda, few available studies done on ADR have shown that a significant number of ADR is prevalent in hospitalized patients [28, 29]. In LMIC, the consequence of ADR is rising fast due to the increased risk factors with limited health care resources [30]. As the HF is increasing in SSA and cardiac drugs can cause a multitude of ADRs, investigation of the prevalence of ADRs and contributing factors in heart failure patients is of utmost importance. In Uganda, there was no published study on ADRs particularly in hospitalized HF patients. Therefore, the present study was planned to determine the prevalence and factors associated with ADRs among heart failure patients hospitalized in MRRH, Mbarara, Uganda.

## Methods

### Study setting and period

This study was conducted at MRRH medical and pediatric ward from 1st November 2021 to 31st January 2022. MRRH is a 600-bed tertiary hospital and is the largest referral center in southwestern Uganda, 280 km far from the capital Kampala. The hospital serves a population of over four (4) million people in its catchment area comprising 13 districts of southwestern Uganda (Mbarara, Sheema, Bushenyi, Rwampara, Kazo, Sheema, Ntungamo, Kiruhura, Ibanda, Buhweju, Rubirizi, Mitooma, Isingiro districts), and the neighboring countries including Burundi, DRC, Rwanda, and Tanzania.

The Medical patient ward is comprised of 50 beds with an estimated monthly admission of 300 patients. Inpatient care is given at this hospital, for cardiovascular patients including heart failure, atrial fibrillation, hypertensive, ischemic heart disease, and other CV causes. The estimated monthly admission of heart failure patients to the adult medical and pediatric ward was approximately 30 and 7, respectively.

### Study design

A prospective observational study was conducted among hospitalized HF patients.

### Study population

All HF patients who were hospitalized at MRRH (adult medical ward and pediatric ward) during the study period, who was diagnosed with HF and willing to participate in the study were the study population. We excluded patients who were critically ill patients, who were not able to respond, and those who were discharged or dead in less than 48 hours of admission.

### Sample size determination and sampling technique

The sample size was calculated using a single proportion formula;

n = Z^2^ p(1-p) / w^2^.

The prevalence of ADR among HF patients in a previous study in Ethiopia was 7.6% [31]. Since the study settings were similar, we used 7.6% as the expected prevalence (p) of ADR in hospitalized heart failure patients, with a 0.05 significance (alpha) level at a 95% confidence interval (CI).

*p* = 7.6% w = 0.05 z = 1.96 (at CI of 95%).

Using the above formula the number of patients included in the study was = 108, 10% of contingency for incomplete data or withdrawal from the study was added;

= (10% * 108) = 11.

Target sample size to be interviewed = (108 + 11) = **119 participants**.

A review of the admission record to the medical and pediatric inpatient department for 3 consecutive months (January, February, March 2021), at MRRH, was done. An average of 30 Heart failure patients per month were admitted to the medical inpatient ward, whereas an average of 7 HF patients were admitted to the pediatric ward.

### Sampling technique

A consecutive sampling technique was used during the study period until the sample size was achieved. The data collection was continued for 3 months until the required sample size was achieved (November 2021 – January 2022).

### Data quality control technique

The research team included clinical pharmacists and physicians (senior residents from the internal medicine and pediatrics department). The pharmacists including the principal investigator collected the data, did the vital signs, and assessed suspected possible ADRs based on the pharmacologic effect of the initiated medications while the senior residents and principal investigator (EAS) discussed and confirmed the suspected ADRs and ruled out other possible causes. Training on data collection protocols and ethical considerations was given prior to the study commencement. The questionnaire was pre-tested among 10 hospitalized HF patients at MRRH that was used prior to the actual data collection. The principal investigator (EAS) was actually involved in the data collection and checked the data completeness daily throughout the data collection process.

### Data collection method

#### Data collection tool

Questionnaire-based interviews were conducted amongst eligible participants to obtain participants’ baseline socio-demographic, past medical history, medication use (including over-the-counter and herbal medicines), social drug use (alcohol and tobacco use), and any known drug allergies. A data collection form was used to obtain data from patients’ medical files. The patient’s vital signs were taken daily and recorded. Laboratory and diagnostic investigations and current medication use were recorded daily. The above-mentioned information was collected by the Research Assistant (pharmacist).

ADR was defined according to Edwards and Aronson’s definition of ADR as presented above [15]. The known adverse reaction profile of each drug was evaluated based on Ugandan Clinical Guidelines (UCG, 2016), and Up-To-Date (2019) version 3.12.0.44 ADRs were first suspected when there is a relationship between the time of drug administration and the onset and course of the adverse reaction while excluding other potential causes.

The probability of ADR was determined using a standard causality assessment tool, Naranjo adverse drug reaction probability scale [32]. Body systems affected by the ADRs were classified using the International Statistical Classification of Diseases for Mortality and Morbidity Statistics (ICD-11 MMS) [33].

The severity of ADR was determined using a modified Hartwig and Siegel severity assessment Scale [34]. The Preventability of ADR was assessed using Schumock and Thornton preventability scale  [35]. The PI (EAS) classified the medications implicated in the suspected ADRs according to the WHO-Anatomical Therapeutic Chemical (ATC) classification [36], whereas Lexicomp software was used to detect potential drug-drug interaction. DDI was recorded as clinically significant when the interaction was rated as C, D, and X as per the Lexicomp drug interaction checker. The above-mentioned tools are standard to assess ADR and valid to use in our study based on previous studies in a similar setting.

#### Data collection process

All patients who presented to the medical and pediatric patient with a diagnosis of HF were subjected to a preliminary screening tool and assessed for eligibility as potential study participants. Data was collected every week from Monday to Saturday, for 3 consecutive months from 1st November 2021 to 31st January 2022.

The research assistant and the principal investigator enrolled patients as study participants upon voluntarily consent to participate in the study by writing. The data collection was conducted always after the routine medical ward round. The study’s aim was explained upon enrollment.

At admission, a detailed history was obtained and a physical examination, by the senior resident physician, was done to identify suspected community-acquired ADRs. To identify the hospital-acquired ADRs, all patients were reviewed daily until discharge, and medications taken were recorded by the research assistants and the PI. The patient’s vital signs were recorded daily from the medical file if done by the routine medical team and if not, it was taken by the researcher to monitor the course of ADR The senior resident did a physical assessment, reviewed the system and gave a clinical opinion on the suspected ADR, and excluded other possible causes for the reaction. When both the principal investigator and the resident physician were confirmed as ADRs, then Naranjo’s scale was used to assess the probability of the suspected ADRs. For any signs and symptoms of ADR, duration, suspected drug, and any drugs used to treat the reaction, were recorded. Assessment of the ADR continued during the ward stay till the patient was discharged.

#### Identification and characterization of suspected ADRs

In this study, we defined liver injury as an increase of AST or ALT value of at least 2 times the upper limit normal. CNS toxicity meant any nightmares, dizziness, insomnia, or lack of concentration. Renal dysfunction was defined as eGFR decline to less than 60 mL/min/1.73 m2 or any increase of serum creatinine by 0.3 mg/dL from baseline or reaching 1.5 mg/dL. Hypotension was defined as systolic blood pressure of < 90 mmHg and diastolic blood pressure of < 60 mmHg. Systolic hypotension was defined as blood pressure of < 90 mmHg while diastolic blood pressure of > 60 mmHg. Constipation was defined as no bowel movement for at least 72 hours or less than three bowel movements per week with any two of the following features. Diarrhea was defined as three or more loose stools within a day (24 hours). Polypharmacy is the use of five or more medications daily by an individual at a time for one or more disease condition.

### Data analysis

All the statistical data analysis was carried out using Statistical Package for Social Sciences (SPSS), version 21 (SPSS Inc., Cary, NC, USA). Descriptive analysis of socio-demographic, clinical, and drug-related variables was presented using median with interquartile range and percentages (%).

The prevalence of ADR among hospitalized heart failure patients was calculated by; dividing the number of patients who had an ADR (at the time of enrolment and during the period of hospital stay) by the total number of patients studied and expressed as a percentage (%). The total person-days were the summation of the hospital stay of all the patients who were followed up in the study. Incidence was calculated by dividing the total new ADR incidents by the total person-days. Univariate and multivariate logistic regression was employed to determine the independent factors associated with ADRs. Variables with *p* < 0.25 in the univariate analysis were included in the multivariate logistic regression. In the multivariate model, *P* values < 0.05 were considered statistically significant.

## Results

### Recruitment and socio-demographic characteristics of participants

Overall, 123 patients were approached; of whom 3 patients were unwilling to consent. Later on, 2 patients were discharged less than 48 hours after enrollment and a total of 118 heart failure patients were included in the final analysis.

The Median (IQR) age of the patients’ was 43 (20.75, 69.25). Forty-eight (40.7%) were elderly patients. Over two-thirds (72, 61%) of the study patients were females (Table 1).

**Table 1:** The socio-demographic characteristics of hospitalized heart failure patients at MRRH, Southwestern Uganda from November 2021 – to January 2022

Variables	Categories	Frequency (%)
Age Median (IQR): 43 (20.75–69.25)	≤18	26 (22)
	19–59	48 (40.7)
	≥60	44 (37.3)
Sex	Male	46 (39)
	Female	72 (61)
Educational status	No formal Education	53 (44.9)
	Primary	53 (44.9)
	Secondary and above	12 (10.2)
Occupation	Unemployed	42 (35.6)
	Self – employed	71 (60.2)
	Employed	5 (4.2)
History of Alcohol Use		33 (28.0)
History of Smoking		19 (18.1)

*IQR Inter quartile range

### Clinical and medication use characteristics

**Fig. 1:**
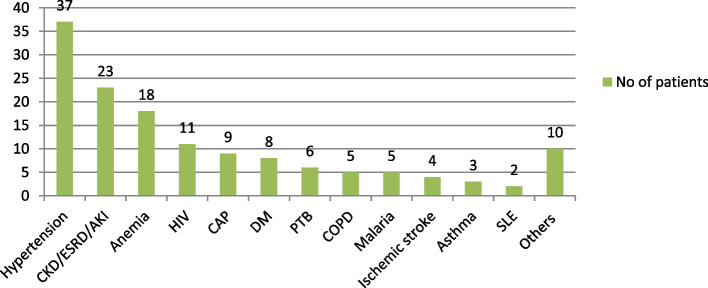
Common comorbid conditions among hospitalized heart failure patients at MRRH, Southwestern Uganda from November 2021 to January 2022

Almost half (56, 47.5%) of the patients were newly diagnosed with heart failure. Thirty-two (27.1%) patients stayed more than 11 days in the hospital. The majority (93, 78.8%) of the patients had at least one comorbid condition (Table 2). Hypertension was the most common (37, 31.4%) comorbid condition followed by kidney disease (23, 19.5%) (Fig. 1).

**Table 2:** The Clinical characteristics and medication use of hospitalized heart failure patients at MRRH, Southwestern Uganda from November 2021 to January 2022

Variables	Categories	N (%)
Duration of the HF	New	56 (47.5)
	Known	59 (52.5)
Previous hospital admission		78 (66.1)
Length of hospital stay (days) Median (IQR): 8 (6–10)	≤5	27 (22.9)
	6–10	59 (50)
	≥11	32 (27.1)
Comorbidity		93 (78.8)
Number of comorbidities (*N* = 93)	One (1)	56 (60.2)
	Two (2) and above	37 (39.8)
Counseling on medication use		107 (90.7)
OTC use within the past 4 weeks		42 (35.6)
Herbal use within the past 4 weeks		52 (44.1)
Poly-pharmacy		75 (63.5)
Significant drug-drug interaction		79 (66.9)
Treatment affordability		18 (15.3)

*Others: Alcoholic liver disease, Hypothyroidism, Thyrotoxicosis, *DVT* Pharyngitis, *ILD* Cholecystitis, *PUD* Cellulitis, Vitamin D deficiency

**Fig. 2:**
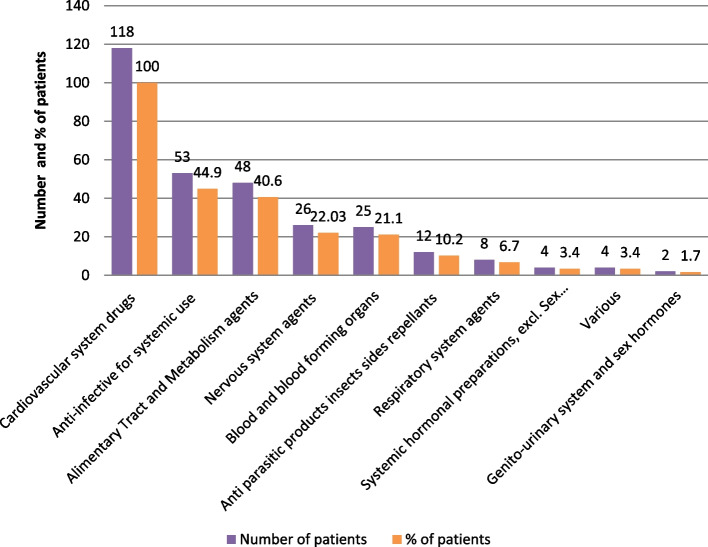
Common medications used among hospitalized Heart failure patients at MRRH, Southwestern Uganda from November 2021 to January 2022

All patients were using at least one cardiovascular agent. Anti-infective agents were used by 53 (44.9%) (Fig. 2). Two-thirds (75, 63.5%) of the patients were on poly-pharmacy and 79, (66.9%) incurred a significant drug-drug interaction among their medications (Table 2).

### Causality assessment of ADRs

Out of a total of 164, ADRs identified, over two-thirds (118, 71.9%) were rated as probable, while 33 (20.1%) and 13 (8%) were possible and definite, respectively.

### Prevalence and incidence of adverse drug reactions among HF patients

**Fig. 3:**
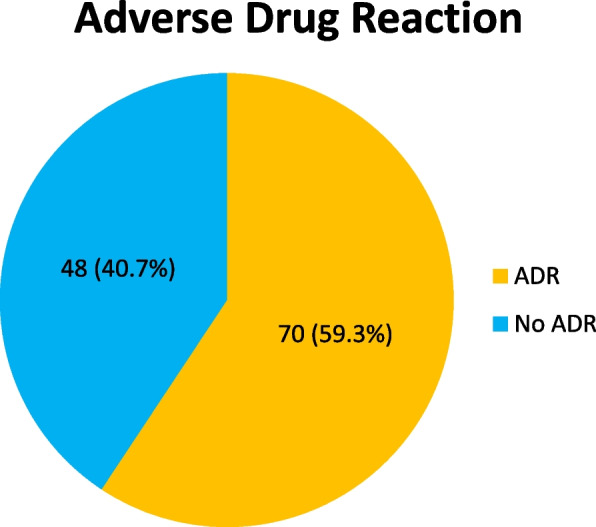
Prevalence of ADR among hospitalized HF patients at MRRH, Southwestern Uganda from November 2021 to January 2022

Seventy out of the 118 patients had at least one ADR at enrollment and during their hospital stay, giving a prevalence of 59.3% (95% CI: 50.8–67.8%) (Fig. 3). Eighteen patients (25.7%) had one, 23 (32.8%) had two, 16 (22.8%) had three, and 13 (18.6%) had four incidents of ADRs.

The incidence rate of ADR was estimated to be 106 ADRs/ 1000 person-days (Table 3). On average 1000 patients would incur 106 (10.6%) incidents of ADR daily during follow-up.

**Table 3:** Incidence of ADR among HF patients at MRRH, Southwestern Uganda from November 2021 to January 2022

Variables	Frequency
Total number of ADRs	164
Total new ADR incidents	107
Total hospital days	1011
ADRs/ persons day × 1000	106 ADRs/ 1000 person-days

### Types of adverse drug reactions

#### Severity and preventability of ADRs

**Fig. 4:**
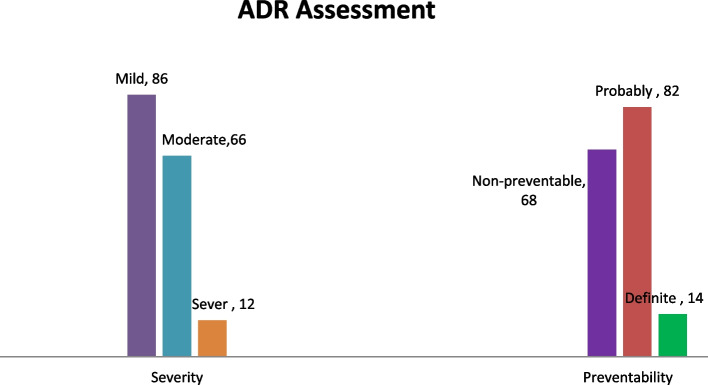
Severity and preventability of ADRs among hospitalized HF patients at MRRH, Southwestern Uganda from November 2021 to January 2022 (*n* = 164)

Over half (86, 52.4%) of the ADRs were mild and 14 (8.5%) were definitely preventable (Fig. 4).

#### Types of adverse drug reactions based on the body systems affected

Over a quarter (45, 27.5%) of the ADRs affected the gastrointestinal system. The nervous system (32, 19.5%) and Endocrine and metabolic system (30, 18.3%) were the second and third most commonly affected body systems respectively. Hyponatremia (21) was the commonest specific ADR identified followed by hypotension (19) and dizziness (15) (Table 4).

**Table 4:** Types of ADRs detected and the body system affected among hospitalized heart failure patients at MRRH, Southwestern Uganda from November 2021 to January 2022 (*n* = 164)

S.no	Type of ADR	N (%)	Specific ADRs with frequency
1	GI	45 (27)	Constipation (12), Nausea (8), Abdominal pain (7), Vomiting (5), Nausea and vomiting (3), Diarrhea (3), Loss of appetite (1), Sore throat (1), Gastritis (3)
2	Nervous system	32 (19.5)	Dizziness (15), Headache (9), Insomnia (3), altered mental status (2), Confusion (1), Body weakness (3), Pain at the injection site (1), Tremor (2)
3	Endocrine and metabolic	30 (18.3)	Hyponatremia (21), Hypokalemia (5), Hypochloremia (2), Hyperglycemia (1), Hypoglycemia (1)
4	CVS	25 (15.2)	Hypotension (19), Reflex tachycardia (1), Systolic hypotension (2), atrial tachycardia (1)
5	Respiratory	8 (4.9)	Dry cough (6)
6	Ocular	6 (3.6)	Blurred vision (6)
7	Otic	5 (3.1)	Tinnitus (4), Reduced hearing (1)
8	Hematologic	5 (3.1)	Increased INR (5)
9	Renal	5 (3.1)	Increased Cr (5)
10	Hepatic	2 (1.2)	Increased liver enzyme (2)
11	Hypersensitivity	1 (0.6)	Anaphylaxis (1)

### Drugs implicated in the ADRs

Cardiovascular drugs were shown to be the drug class most frequently (119, 72.5%) associated with ADRs; Furosemide alone contributed to 57 ADRs, which included 10 of the constipation and all the electrolyte disorders (28). Anti-infective agents (34, 20.7%) were the second most common suspected culprits of ADRs; ceftriaxone was implicated in 11 ADRs (Table 5).

**Table 5:** Causative agents of the ADRs among hospitalized heart failure patients at MRRH, Southwestern Uganda from November 2021 to January 2022

ATC class	Drug	ADR and frequency
Alimentary Tract and Metabolism agents (4)	Bisacodyl (1)	Diarrhea (1)
	Glibenclamide and insulin (1)	Hypoglycemia (1)
	Metformin (2)	Gastritis (2)
Blood and blood-forming organs (8)	Warfarin (5)	increased INR (5)
	Aspirin (2)	Gastritis (2)
	Iron sulfate (1)	Abdominal pain (1)
Cardiovascular system drugs (119)	Digoxin (20)	Blurred vision (6), tinnitus (2), Abdominal pain (3), Confusion (1), Atrial tachycardia(1), Nausea and vomiting (2), vomiting (1), altered mental status (2), tremor (2)
	Bisoprolol (2)	Arrhythmia (1)
	Enalapril (4)	Systolic hypotension (1), Dry cough (3)
	Furosemide (57)	Constipation (10), Hypotension (7), Hyponatremia(21), Hypokalemia(5), Hypochloremia (2), dizziness (10), Nausea (1), Systolic hypotension (2),
	Furosemide, bisoprolol, enalapril (8)	Hypotension (8)
	Furosemide, carvedilol (4)	Hypotension (4)
	Furosemide, Digoxin (3)	Dizziness (2), Abdominal pain (1)
	Carvedilol (2)	Hypotension (2)
	Captopril (2)	Hypotension (1), dry cough (1)
	Nifedipine (13)	Headache (9), Hypotension (3), reflex tachycardia (1)
	Valsartan (1)	Dry cough (1)
	Losartan (4)	Dry cough (3), Increased Cr (1)
Systemic hormonal preparations, excl. Sex hormones and insulins (2)	Carbimazole (1)	Sore throat (1)
	Prednisolone(1)	Hyperglycemia (1)
Anti-infective for systemic use (34)	Ampicillin (2)	Nausea (2)
	Azithromycin (1)	Abdominal pain (1)
	Benzathine Penicillin (2)	Pain at the injection site (1), Anaphylaxis (1)
	Ceftriaxone (11)	Dizziness (6), Diarrhea (1), Nausea (3), Vomiting (1)
	Ciprofloxacin (1)	Nausea (1)
	Gentamicin (3)	Diarrhea (1), vomiting (2)
	Levofloxacin (1)	Tinnitus (1)
	RHZ (3)	Increased liver enzyme (2), tinnitus (1),
	Ethambutol (1)	Increased Cr (1)
	(TDF/3TC/DTG) (3)	Body weakness (3),
	TDF (3)	Increased Cr (3)
	DTG (3)	Insomnia (3)
Anti-parasitic products, insects sides repellants (4)	Metronidazole (2)	Loss of appetite (1), Nausea (1)
	Artesunate (2)	nausea and vomiting (1), Abdominal pain (1)
Nervous system agents (3)	Morphine (1)	Constipation (1)
	Tramadol (1)	Nausea and Vomiting (1), constipation (1)
	Pregabalin (1)	Dizziness (1)

### Factors associated with ADR among HF patients

#### Univariate logistic regression

A total of 15 independent factors were analyzed in univariate logistic regression. Among those, age ≥ 60 (COR 3.30 [1.11–9.83] at 95% CI, *p* = 0.032), education, duration of the disease, previous hospital admission, length of hospital stay, OTC use within the previous 4 weeks, herbal use within the previous 4 weeks (COR 5.7 [2.45–13.27] at 95% CI, *p* = 0.001), poly-pharmacy (COR 7.31 [3.16–12.92] at 95% CI, p = 0.001) and drug-drug interaction (COR 14.13 [5.49–21.34] at 95% CI, p = 0.001) were qualified for multivariate logistic regression analysis at *P*-value of < 0.25 (Table 6).

**Table 6:** Factors associated with ADRs among hospitalized heart failure patients at MRRH, Southwestern Uganda from November 2021 to January 2022

Variables	Categories	ADR (NO) 48 (40.7%)	ADR (YES) 70 (59.3%)	COR (95%CI)	P-value	AOR (95% CI)	P-value
Age*	≤18	11 (42.3)	15 (57.4)	1		1	
	19–59	29 (60.4)	19 (39.6)	0.48 [0.18–1.27]	0.138	0.15 [0.03–0.35]	**0.013**
	≥60	8 (18.2)	36 (81.8)	3.30[1.11–9.83]	**0.032**	1.97 [0.45–9.13]	0.384
Gender	Male	19 (41.3)	27 (58.7)	1			
	Female	29 (40.2)	43 (59.8)	1.04 [0.94–2.21]	0.912	#	#
Education *	No formal Education	20 (37.7)	33 (62.3)	8.25[1.64–41.55]	**0.011**	2.02 [0.24–16.8]	0.515
	Primary	18 (33.9)	35 (66.1)	9.7[1.9–49.18]	**0.006**	2.26 [0.29–17.41]	0.433
	Secondary and above	10 (83.3)	2 (16.7%)	1		1	
Occupation	Unemployed	18 (42.9)	24 (57.1)	2 [0.3–13.24]	0.472	#	#
	Self – employed	27 (38.1)	44 (61.9)	2.44 [0.38–15.58]	0.344	#	#
	Employed	3 (60)	2 (40)	1			
Disease Duration *	New	26(46.4)	30 (53.6)	1		1	
	Known	22 (35.5)	40 (64.5)	1.58 [0.75–3.30]	0.228	1.15 [0.36–3.67]	0.810
Previous hospital admission*	Yes	27 (34.6)	51 (65.4)	2.09 [0.96–4.54]	0.063	2.09 [0.68 - 6.44]	0.202
	No	21 (52.5)	19 (47.5)	1		1	
Length of hospital stay*	≤5	18 (66.7)	9 (33.3)	1		1	
	6–10	20 (33.9)	39 (66.1)	3.9 [1.49–10.23]	**0.006**	0.71 [0.16–3.28]	0.670
	≥11	10 (31.2)	22 (68.8)	4.4 [1.47–13.15]	**0.008**	0.64 [0.11–3.67]	0.615
Counseling	Yes	44 (41.1)	63 (58.9)	0.82 [0.23–2.97]	0.76	#	#
	No	4 (36.4)	7 (63.6)	1			
Comorbidity	Yes	39 (41.9)	54 (58.1)	0.78 [0.31–1.95]	0.592	#	#
	No	9 (36)	16 (64)	1			
OTC use within the past 4 weeks*	Yes	14 (33.3)	28 (66.7)	1.62 [0.74–3.5]	0.229	1.41 [0.45–4.39]	0.558
	No	34 (44.7)	42 (55.3)	1			
Herbal use within the past 4 weeks*	Yes	10 (19.2)	42(80.8)	5.7 [2.45–13.27]	**0.001**	3.07 [1.01–9.32]	**0.048**
	No	38 (57.6)	28(42.4)	1			
Smoking history	Yes	6 (31.5)	13 (68.5)	1.6 [0.56–4.55]	0.381	#	#
	No	42 (42.4)	57 (57.6)	1			
Alcohol use history	Yes	11 (33.3)	22 (66.7)	1.54 [0.67–3.58]	0.313	#	#
	No	37 (43.5)	48 (56.6)	1			
Poly-pharmacy*	Yes	18 (24)	57 (76)	7.31 [3.16–12.92]	**0.001**	8.7 [2.4–15.77]	**0.001**
	No	30 (69.8)	13 (30.2)	1		1	
Drug–Drug interaction*	Yes	17 (21.5)	62 (78.5)	14.13 [5.49–21.34]	**0.001**	6.06 [2.79–12.5]	**0.004**
	No	31 (79.5)	8 (20.5)	1			

* *< 0.25, bold- < 0.05, # - N/A, COR - Crude odd ratio, AOR - Adjusted odd ratio, CI – Confidence interval, OTC – over the counter*

#### Multivariate logistic regression

Accordingly, the Multivariate logistic regression showed that younger adults aged 19–59 were less likely associated with ADRs; (AOR 0.15 [0.03–0.35] at 95% CI, *p* = 0.013). Patients who had been using herbal drugs within 4 weeks before admission were 3 times more likely to ADRs than those who did not use; (AOR 3.07 [1.01–9.32] at 95%CI, *p* = 0.048). Additionally, patients who were taking more than 5 medicines during hospital stay were 8.7 times more likely to experience ADRs; (AOR 8.7 [2.4–15.77] at 95% CI, *p* < 0.001). ADR among HF patients was also significantly associated with an independent factor of a drug-drug interaction; (AOR 6.06 [2.79–12.5] at 95% CI, *p* = 0.004) (Table 6).

## Discussion

In the current study, almost two-thirds (59.3%) of the HF patients had at least one ADR during their hospital stay. The current prevalence is comparable with 53.2% [37] and 67% [23] in India and 69% in Indonesia [38].

However, our finding of prevalence among hospitalized HF patients is higher than in previous studies done in high and middle-income countries including 8.6% in Italy [39], 7.74% in UAE [17], and 24.2% in Iran [40]. The current higher prevalence may be attributed to our prospective study design and ADR assessment method. Accordingly, we detected ADRs at enrollment, reviewed patient records, interviewed patients, did the physical examination, and followed up until they were discharged, which provided ample time to detect the ADRs. In contrast, some of the former studies [17] were done observationally and some were solely based on patient records, retrospectively [39].

In addition, the lack of active pharmacovigilance in our setting based on a study done in 2018 [41] has contributed to the increased prevalence of ADRs, As the reported ADRs help to avoid known risk factors and monitor patients. Moreover, more than half (59.3%) of this study’s patients were either elderly or pediatric, who are known for being vulnerable to ADRs. Also, the majority (78.8%), of our study participants had at least one comorbid condition that predisposes them to receive multiple medications and leads to ADRs. The incidence of new ADR was 106 ADRs/ 1000 person-days. If 1000 HF patients were followed up in a day, on average 106 (10.6%) would incur an ADR.

The Naranjo causality scale rated over two-thirds (71.9%) of the ADRs as probable. The probable causality was considerably higher than those in other studies including, 58.3% in UAE [17], 56.7% & 18.4% in India [23, 37], and 33.3% in Iran [40]. Additionally, Lupitaningrum et al. reported 41.9% of probable ADRs in Indonesia among hospitalized HF patients [38]. The deviation with some of the studies could possibly be because of the method used to assess the causality, which was the WHO classification of causality [37, 40]. In the present study, however, daily monitoring of patients, laboratory investigation review, and physical assessment added an objective proof for the ADRs and increased the probability of ADRs.

GI system was the most frequently (27.5%) affected system by ADRs, followed by the nervous system (19.5%), Endocrine & metabolic systems (18.3%). These results are in line with previous findings that had shown the GI system as the commonly affected system among hospitalized HF patients [38, 39, 42].

The present study revealed that electrolyte imbalance, majorly hyponatremia (12.8%), is the most common specific ADR followed by hypotension and dizziness. Electrolyte imbalance was also noted as one of the frequent ADRs among hospitalized HF patients by Catananti et al. [39]. In our study, hypotension (11.5%) was mainly caused by the combination of anti-hypertensive agents and IV diuretics. This was in line with previous studies [23, 37, 43].

Over half (52.4%) of the ADRs were mild, while 40.2 and 7.3% were moderate and severe, respectively. The proportion of severe ADRs in this study was comparable with previous studies of 10.9% & 13.5% in India [23, 37], 9.1% in UAE [43] and 4.9% in Italy [39].

Additionally, this study revealed that over half (58.5%) of the ADRs were preventable: 50% were probably preventable, whereas 8.5% were definitely preventable. The preventable nature of the ADRs calls for attention by the health care team involved in prescribing and following up with patients. It accounts for demonstrating prevention strategies among at-risk patients for ADRs.

Patients at high risk for ADR, including patients with comorbid conditions, who are on poly-pharmacy, elderly, and pediatrics, need special attention while prescribing, monitoring, and assessing them. It is fact that preventable ADRs are a significant burden to health care among hospitalized patients [44]. The proportion of preventability in the current study is comparable with previous studies done among hospitalized HF patients, in which preventability was 65.9% [37] and 40% [42].

Almost three-quarters (72.5%) of the ADRs were caused by cardiovascular drugs. Furosemide, a diuretic agent, alone contributed to almost half (48%) of the ADRs caused by cardiovascular drugs. Previous findings showed that specific drug commonly implicated in ADRs among hospitalized HF patients was digoxin & furosemide [37], and Bisoprolol [38, 43].

The identification of associated factors for ADRs helps to identify the most susceptible patients who require close monitoring of drug therapy [45]. Our study showed that ADR was less likely associated among younger adults (19–59 years old) by 85%, compared to the age group less than 18 (pediatrics). This finding is supported by a number of studies that revealed that age being very old or very young compared to younger adults, were susceptible to ADRs [23, 37, 46]. This may be is explained by the fact that there is a pharmacokinetic and pharmacodynamics change among elderly and pediatric populations [47].

The present study reported that patients who were taking more than 5 medicines during hospital stay were almost nine times more likely to experience ADRs. Former studies done among hospitalized HF patients [23, 43, 48] were in agreement with the current finding that poly-pharmacy was a significant risk factor and every additional treatment had an increased risk of increasing ADR by 8.6–9% [47, 49]. Poly-pharmacy has been associated with an increased risk of drug interaction and ADRs [48, 50]. This calls for attention to the use of medications among HF patients by providing only the necessary medications and avoiding the overuse of multiple medications, which might lead to ADRs.

Patients who had used herbal drugs in the 4 weeks before admission were as well 3 times more likely to experience an ADR than those who did not. The safety and effectiveness of herbal drug use are not yet assured and the interaction with conventional medicine leads to ADRs [51].

Patients who had a significant drug-drug interaction (DDI) had six times the odds of ADRs as revealed in former studies that DDI was significantly associated with ADRs [50, 52]. The synergistic effect of the DDI may attribute to ADRs. Clinical pharmacists have a significant role in detecting and preventing DDI-related ADRs. Studies have reported a decreased occurrence of ADR and drug-related problems as clinical pharmacists are engaged in medication review as part of the multidisciplinary team for optimizing patient safety [53, 54].

### Strength and limitation

This study provided detailed information on the types of ADR and associated factors among HF patients. There is a paucity of literatures done in this research area in LMIC. Hence, the present study will help to fill the unprecedented evidence gap in our setup and to come up with a solution for the problems in the future perspective. Moreover, this study describes the epidemiological data from an African setting. Since most of available literatures were conducted in developed countries, this study gives the highlight of the problem in African setting.

The prospective nature of the study enabled us to gather complete information daily and assess and record the problem directly from the patients. Moreover, the data was collected by the PI (clinical pharmacy Masters Student), with the assistance of resident physicians, which increases the quality and accuracy of the data.

Despite the strength, our study had some limitations. For one, the study was single centered and conducted in a hospital serving referred patients who have severe illnesses and more comorbidities, which makes the finding slightly difficult to generalize to a larger population. In addition, objective measurements (laboratory investigation results) had a great impact in showing the disease progress, response to treatment, and ADRs caused by the initiated drug. Clinical examination and patient reports were mainly used as a method used to identify suspected ADRs.

## Conclusion

The current study showed that almost two-thirds of the hospitalized HF patients experienced at least one ADR during their hospital stay, whereas more than one in ten patients experienced a new ADR per day during hospitalization. Over two-thirds of the ADRs were rated as probable. The gastrointestinal system, nervous system, and endocrine and metabolic systems were the top three most frequently affected systems. Over half of the ADRs were mild whereas almost two-thirds were preventable. Herbal use within 4 weeks prior to admission, poly-pharmacy, significant drug-drug interaction, and being in the age bracket of 19–59 was shown to be factors significantly associated with ADRs among hospitalized HF patients.

Based on our findings, we recommend the health care team working at MRRH and other health facilities, actively assess ADRs and intervene in the preventable ADRs before they occur. Since more than half of the ADRs in our study were preventable, the health care team can cautiously work on the gaps identified by this study to improve the outcome of the patient such as monitoring the drugs involved, assessing drug interactions, and use of preventive agents, thus reduce unnecessary expenses and improve treatment outcomes.

Patients need to be counseled about the appropriate use of herbal medicine, medication adherence, expected side effects of the drugs, and regular follow-up by the health care team as part of the treatment.

Additionally, we believe including clinical pharmacists as part of the disciplinary team will help to tackle this problem as they check drug interactions, assess medication use, and monitor signs of ADRs. Stakeholders and the ministry of health can integrate this program, nationwide.

### Data sharing statement

The datasets used and analyzed during the current study are available from the corresponding author on reasonable request.

## Abbreviations

ACEI: Angiotensin-converting enzyme inhibitor
ADE: Adverse drug events
ADR: Adverse drug reaction
ARB: Angiotensin receptor blocker
ARNI: Angiotensin receptor-Neprilysin inhibitor
ATC: Anatomical therapeutic chemical classification
CI: Confidence interval
CVD: Cardiovascular disease
DDI: Drug-drug interaction
eGFR: Estimated glomerular filtration rate
GI: Gastrointestinal
HF: Heart failure
ICD: International Statistical Classification of Diseases
LMIC: Low and middle-income countries
MRA: Mineralocorticoid Antagonist
MRRH: Mbarara Regional Referral hospital
NCD: Non-communicable disease
OTC: Over the counter
SSA: Sub-Saharan Africa
UAE: United Arab Emirates
WHO: World Health Organization
